# The organophosphate pesticide -OP- malathion inducing thyroidal disruptions and failures in the metamorphosis of the Senegalese sole, *Solea senegalensis*

**DOI:** 10.1186/s12917-019-1786-z

**Published:** 2019-02-11

**Authors:** Juan B. Ortiz-Delgado, Victoria Funes, Carmen Sarasquete

**Affiliations:** 1 Instituto de Ciencias Marinas de Andalucía-ICMAN, CSIC Campus Universitario Río San Pedro, 11510 Puerto Real, Cádiz Spain; 2 IFAPA, Centro el Toruño, Junta de Andalucía, Camino Tiro de Pichón s/n, 11500 El Puerto de Santa María, Cádiz Spain

**Keywords:** Eye, Malathion, Metamorphosis, Osteocalcin, Proliferation, Proteins, Skeleton, Senegalese sole, Thyroid, Transcripts

## Abstract

**Background:**

Organophosphate pesticides-OP-, like malathion, can alter the normal functioning of neuro-endocrine systems (e.g., hypothalamus-pituitary-thyroid-HPT- axis), and to interfere on the thyroidal homeostasis. Through direct interactions with thyroid receptors, an/or indirectly via up-stream signalling pathways, from the HPT axis (i.e., negative feedback regulation), malathion possess the ability to affect integrity of thyroidal follicular tissue, and it can also block or delay its hormonal functioning. This insecticide can alter the majority of the ontogenetic processes, inducing several deformities, and also provoking decreases in the growth and survival patterns. The present study has been performed to determine the sublethal effects of malathion during the first month of life of the Senegalese sole, *Solea senegalensis*, and it is mainly focused on the metamorphosis phase. Different transcript expression levels (i.e. thyroid receptors, matrix and bone -Gla-proteins) and immunohistochemical patterns (i.e. thyroid hormones, osteocalcin, cell proliferation) have been analysed during the most critical phases of the flatfish metamorphosis, that is, through differentiation of thyroid system and skeletal development, migration of the eye, and further adaptation to benthic behaviours.

**Results:**

In early life stages of the Senegalese sole, the exposure to the highest concentration of malathion (6.25 μg/L) affected to the growth patterns, showing the exposed individuals, a reduction around 60 and 92% of the total length and the dry weigth, respectively. In paralell, a significant reduction of the thyroid follicles (i.e., size and number) it was also been recorded, in a dose-dependent way. Abnormal phenotypes induced in the exposed larvae, did not complete the process of metamorphosis, and displayed several morphological abnormalities and developmental disorders, which were mainly associated with the eye migration process, and with thyroidal and skeletal disorders (i.e., transcriptional and protein changes of thyroid hormones and receptors, and of matrix and bone Gla proteins distribution), that conduced to an inadequate adaptation to the benthic life.

**Conclusions:**

In the Senegalese sole, the majority of the ontogenetic alterations induced by the exposure to malathion were mainly associated to the metamorphosis period, which is a thyroid-driven proccess. In fact, most crucial and transitional ontogenic events, appeared notably disturbed, for e.g., thyroid gland differentiation and functioning, migration of eye, skeletal development and benthonic behaviors.

## Background

The intensive use of pesticides such as insecticides, herbicides, fungicides, acaricides, among others, has led to ubiquitous contamination, being present not only in soils, water-bodies and crops, but also in the atmosphere as fine airborne particles, as it has recently been reported by using an interesting and innovative green technology [1]. One of the most serious consequences of the increasingly use of pesticides is the chemical pollution of freshwater and estuarine ecosystems, which are the ultimate storehouse of their residues [2–4]. Due its high persistence and bio-accumulative properties, these synthetic chemicals affect behavioral and physiological systems of the aquatic inhabitants, particularly those of the fish [5]. Pesticides, even at very low concentrations provoke hazardous impacts on basal metabolisms. These harmful effects are expressed either, in terms of mortality, or by different alterations, such as retardation in growth, development and reproduction, among other physiological processes, depending on the physical and chemical properties of the pesticides, its concentrations, capability of degradation, and the ability of fish to metabolise them [6–8]. Natural and synthetic pesticides are used to kill unwanted organisms in crops, public areas, homes and gardens, and parasites. Many chemicals that have been identified as endocrine disruptors (EDCs) are pesticides, and several different effects linked to endocrine disruptions have been largely reported in invertebrates, fish, reptiles, birds and mammals [9–14]. Among many other environmental EDCs, pesticides can alter the normal functioning of the neuro-endocrine systems (e.g. the thyroid axis), and therefore these xenobiotics can interfere with endocrine homeostasis and dependent-hormonal processes. Besides, pesticide induced thyroid disruptions can severely compromise different developmental processes, growth, fitness and survival of the exposed fish [4, 9, 11, 12, 15]. Disruptions of the thyroid status and functioning can occur through several steps, i.e. in the synthesis, regulation, metabolism, transport and action of the thyroid hormones/THs [16, 17]. Pesticides can affect thyroid signalling by blocking, mimicking, or synergizing to the endogenous hormones, through direct thyroid receptor (TRs) interactions, and indirectly via upstream signalling pathways, by inhibiting hormonal synthesis [13, 18]. In addition, many pesticides possess the ability to affect the thyroid gland integrity itself, such as it has been reported in different fish species [7, 19, 20].

As it is very well known, in vertebrates but most notably in anuran amphibians, and in flatfish species, the complex neuroendocrine-thyroid system is the main key for the progress of the metamorphosis, and this thyroidal axis serves to transduce environmental informations into a set of endocrine and sympathetic responses, that regulate specific alterations of the behavioural, morphological and physiological systems [21–23]. Flatfish metamorphosis culminates with the transition to definitive juvenile phenotype and behavior, and this transitional ontogenetic proccess is previously encompassed by the reorganisation and maturation of the digestive, respiratory, visual (e.g. eye migration) and neural systems, as well as noticeable changes in the skin-pigmentary patterns and skeletal and muscular structures, among other developmenal variations [22–28]. Although the molecular mechanisms and genes involved in the metamorphic processes have still not yet been fully elucidated, currently it is very known that the hypothalamus-pituitary-thyroid (HPT) axis mediates the metamorphosis of flatfish species, just as it does in amphibians [13, 22, 23, 29], among others. Accordingly, thyroid disruptions may alter and compromise the metamorphosis of flatfish, and therefore the viability, fitness and healthy survival of larvae and juveniles. The flatfish metamorphosis is one of the most critical ontogenetic developmental events, and also one of the most sensitive to environmental toxicants (i.e. chemicals, phytochemicals, etc.), and particularly against those endocrine disruptors targeting the thyroid axis [9, 22, 30, 31]. In addition, thyroid hormones (THs) are involved in the most important physiological and ontogenetic processes, such as vision, feeding, behavior, metabolism, growth, pigmentation, ossification and development and differentiation of the cardiovascular, digestive and muscular systems, among others [14, 22, 23, 32]. Furthermore, during the flatfish ontogeny, some of the most critical and sensitive events are especially related with the development and differentiation of the eyes, and craniofacial structures, since they are essential target-organs for avoiding predators, locomotory performance and food intake [22, 31, 33–37]. In vertebrates, the eye and craniofacial developments are complex processes regulated by THs, and treatments with many thyroid disruptors result in malformations of eyes and head, reduced eye-sizes, and disruptions of retinal cell- layers [15, 23, 38]. In this contex, the development and differentiation of photoreceptors occurs through direct regulation of thyroid hormones (THs); and thyroid receptors (TRs) are expressed in the outer nuclear layer (ONL) of the retina, which contains the differentiating photoreceptors [39, 40]. In addition, THs are required for normal skeletal development, and they have a crucial role in osteoblastic differentiation [41, 42]. It has been reported that reduced levels of THs cause growth inhibition of the cranial base of cartilage and bone, resulting in a wide and short face with an underdeveloped mandible [43].

On the other hand, organophosphate pesticides (OPs) are a group of synthetic chemicals, used for many domestic and industrial purposes. Over time, they have been the most commonly used as insecticides, accounting 50% of the global use, and they have been responsible for numerous pesticide poisoning [44]. The non-systemic and wide-spectrum organophosphate insecticide malathion [1,2-Di (ethoxycarbonyl)ethyl O,O-dimethyl phosphoro-dithioate], is one of the earliest organophosphate insecticides developed in 1950 and was first registered for using in 1956, by the United States Department of Agriculture (USDA), and it was regulated (Registration Eligibility Decision, RRD) by the United States Environmental Protection Agency (USEPA) [45]. Despite its ban by the European Commission, since June 2007 (Regulation EC 1376/07,47 07/389), malathion is still widely used in developing countries worldwide provoking harmful environmental problems in coastal areas, because of its runoff from agriculture and urban sources [4, 46, 47]. Under the present legislation, in Europe, the use of the OP pesticides is prohibited and, therefore these pollutants should not be present in the environment. However, in the last years, some data of pollution by OP pesticides have been registered in Mediterranean coastal areas [48, 49]. Pesticides, such as malathion, dichlorvos, quinalphos, and other contaminants have been measured in some costal aquaculture systems [50]. Several studies have reported high malathion concentrations ranging from 2.62 to 105.2 μgL^− 1^ in different ground-water samples, from different worldwide areas [51, 52]. Additionally, the RASFF (Rapid Alert System for Food and Feed) from European Commission reported 13 notifications during 2006 to 2014 of pesticide residues, in fish fillets from Indian areas [53].

In different fish species, the OP malathion has been reported to alter the thyroid system, causing decreased thyrotropin (TSH) synthesis and secretion, as well as reduction of thyroid hormonal levels, which provoked serious disrupting effects in developmental and growth patterns, behaviours, fitness and survival [2, 4, 7, 9, 54]. A review about malathion toxicity and effects on fish (i.e. bioaccumulation, behavioural and neurotoxicant responses, histopathological and haematological alterations, respiratory responses, biochemical parameters, chromosomal damages, etc.) has recently been published [3]. In addition, in a recent study performed at the early life stages of the Senegalese sole, Ortiz-Delgado et al. [55] reported that the exposure to acute concentrations of malathion (i.e. at 72 h, LC50: 22.94 μg/L; LOEC: 3.12 μg/L and NOEC: 1.6 μg/L) altered the notochord and trunk musculature integrity, displaying noticeable changes of the composition of collagen fibers from the perinotochordal connective sheath, and other serious neural and somatic ontogenetic disorders.

In accordance with all of these referenced findings, and from many other existing reports, on the toxicity of malathion and its effects on different fish species, it has been hypothesized that most of the disruptive thyroidal responses induced by the OP- malathion, should alter the thansitional and complexity processes driven by the thyroid-axis; that is the metamorphosis process of this flatfish, and should also alter normal growth pattern, behavior and phenotype, as well as fitness and healthy survival of juveniles. Therefore, a subacute exposure to environmentally-relevant sub-lethal concentrations of malathion during the larval development of the Senegalese sole, *S. senegalensis* is analysed in the present study, by using several biological, biometric, molecular and cell markers. Thus, the aim of this work has been: i) To analyse the subacute effects of sub-lethal concentrations of the OP malathion, administered at the early life stages of the Senegalese sole, and particularly focused during the metamorphosis process, by analysing several disrupting effects targeted on thyroid follicles; eye/retina and skeletal development; and ii) To provide some insights into underlying molecular mechanisms, that are inducing abnormal metamorphosis, due to malathion exposure. To achieve this goal, three concentrations of malathion (1.56, 3.12 and 6.25 μgL^− 1^) have been tested during the first month of larval life (from 4 dph until 30 dph). The growth patterns, and the effects on the eye differentiation and migration, as well as on the thyroid gland ontogeny and on the skeletal development were also analysed and discussed.

## Results

### Growth patterns

**Fig. 1:**
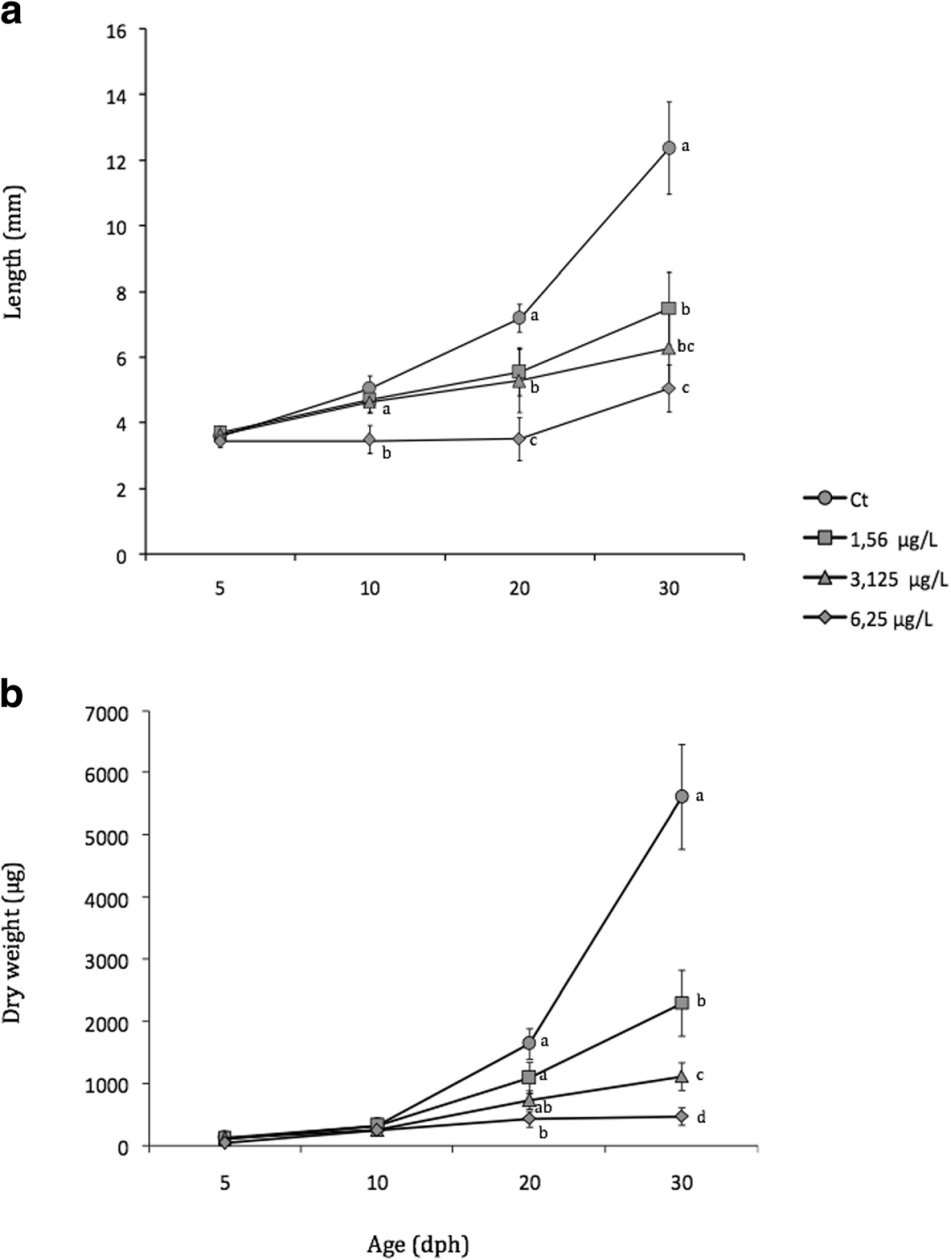
Changes in total length (**a**) and dry weight (**b**) (mean ± SD) of *Solea senagalensis* larvae exposed to different malathion concentrations. Different letters denote significant differences (*P* < 0.05) between control and malathion treatments at the same age

**Fig. 2:**
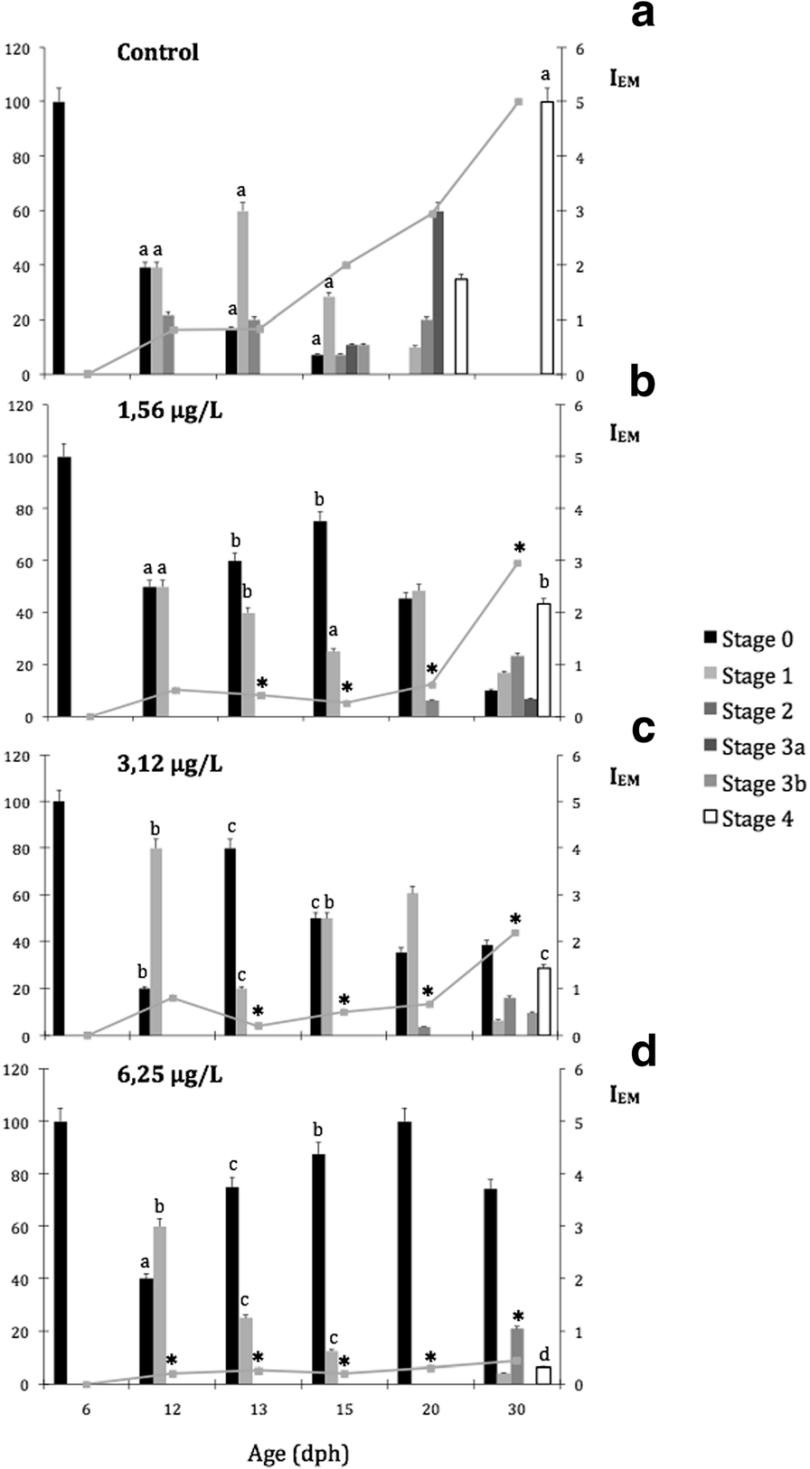
Changes in eye migration of Senegalese sole control larvae (**a**) and exposed to different malathion concentrations (**b** to **d**). Vertical bar show the percentage ± SD of fish at each stage of development. Different superscripts denote significant differences (*P* < 0.05) between control and malathion treatments at an age. Eye migration index (*I*_EM_) is shown as a line plot on each vertical bar chart. Asterisks shown significant differences (*P* < 0.001) between control and malathion treatments at the same age

The growth pattern of the Senegalese sole, during the first month of larval life, in terms of total length (TL) and dry weight (DW) is represented in Fig. 1. The controls showed a typical allometric growth pattern, as well as an adequate larval development, and a normal organogenesis, with progressive and characteristic migration of one of the eyes. The percentage of larvae with correct migration of the eye ranged between 40% (at 20 dph) and 100% (at 30 dph) (Fig. 2). By increasing both the time of exposure and malathion concentrations, significant morphological and ontogenetic changes were registered during the first month of life. In fact, a delayed growth pattern was recorded in parallel with increases of the malathion concentrations tested, being the TL significantly reduced from 20 dph onwards, when larvae were exposed to 1.56 and 3.12 μg/L, and earlier (from 10 dph onwards) in exposures at the highest concentration (at 6.25 μg/L) assayed. At the end of the experimental period (at 30 dph), body length leveled off from 13 mm of TL in controls to approximately 6–8 mm in larvae from all malathion treatments (at 1.56, 3.12 and 6.25 μg/L) (Fig. 1a). On the other hand, the DW was also significantly reduced at 30 dph, with malathion treatments of 1.56 and 3.12 μg/L, and from 20 dph onwards with the highest malathion concentration tested (at 6.25 μg/L), showing a weight reduction up to 90% in exposed larvae at 30 dph, in comparison with controls (Fig. 1b).

The advancement of metamorphosis in terms of the eye migration is represented in Fig. 2. The malathion exposure significantly slowed the metamorphosis, in a time- and dose- dependent manner. At 1.56 and 3.12 μg/L of malathion, statistically significant differences between controls and exposed larvae were registered from 13 dph onwards, whereas at 6.25 μg/L, significant changes were displayed earlier in the larval development (from 12 dph onwards). In controls, at 30 dph, the index of eye migration (*I*_EM=_ 5) was completed and fully normal and presented less advanced metamorphic stages, in a concentration-dependent manner, for those exposed fish (*I*_EM_: 2.95 at 1.56 μg/L; *I*_EM_: 2.19 at 3.12 μg/L and *I*_EM_: 0.44 for 6.25 μg/L) (Fig. 2 a to d).

**Fig. 3:**
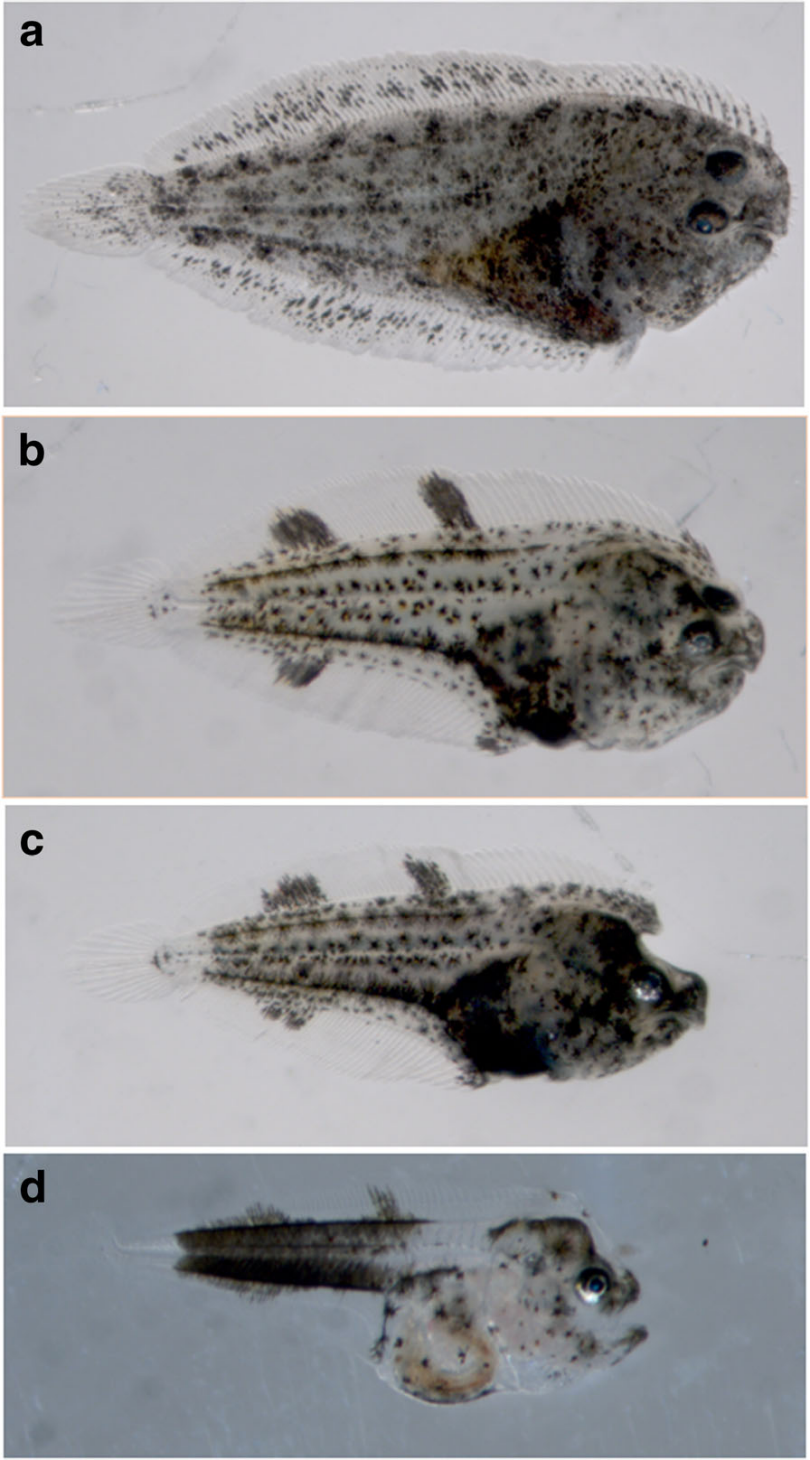
Morphological aspects of Senegalese sole larvae at 30 dph. Note the different phenotypes in control (a) and 1.56 (b), 3.12 (c) and 6.25 (d) μg malathion/l

Regarding the phenotypes, the larvae exposed to malathion did not complete the metamorphosis process and displayed, at the end of experimental period, abnormal morphologies showing eye migration failures and pigmentary disorders (Fig. 3 a to c). Larvae exposed to intermediate concentration (at 3.12 μg/L) showed the lack (bilaterally symmetric), or an incomplete eye migration (at 1.56 μg/L), but displayed morphological characteristics and normal lateralized behaviors, settled with their left-side on the botton, such as it is observed in the controls (Fig. 3 b and c). Most larvae at 6.25 μg/L showed (74%) the metamorphic Stage 0 without signs of eye migrations. These larvae, still planktonic, showed a morphological pattern similar to that observed in pre-metamorphic larvae, with the absence of morphological characteristics or normal lateralized behaviours, which are necessary for the adaptation to benthic- life (Fig. 3d).

### Effects on the thyroid gland development

**Fig. 4:**
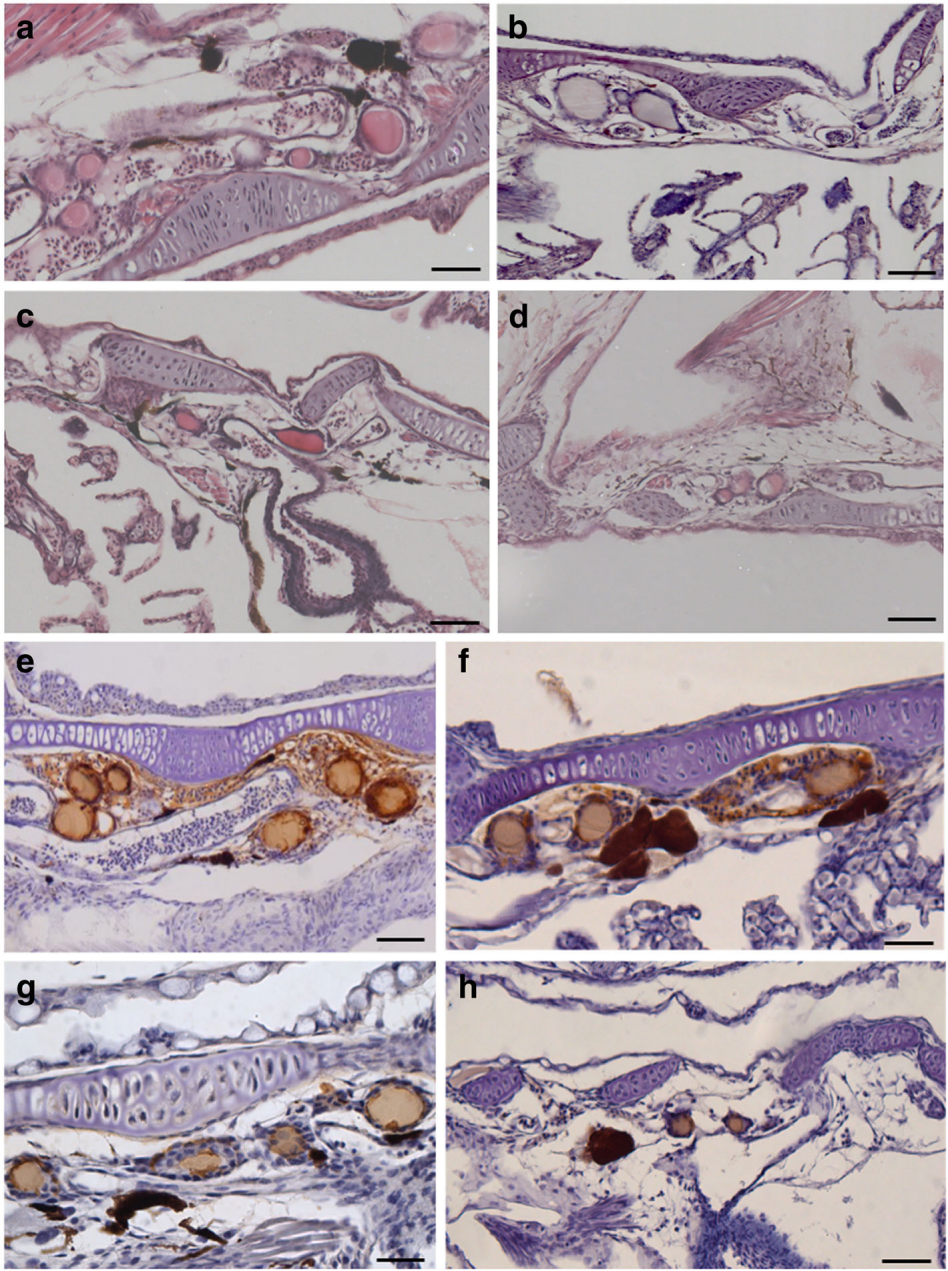
(**a** to **d**) Tissue sections of thyroid gland of Senegalese sole at 30 dph showing thyroid follicles from controls (**a**) and 1.56 (**b**), 3.12 (**c**) and (6.25) malathion exposed larvae: **a** Control specimens showing a typical distribution of thyroid follicles around the ventral aorta. **c** to **d** Thyroid gland from malathion exposed specimens showing a reduction in number, size and epithelial height of thyroid follicles in comparison with controls. **e** to **h** T_4_ immunolocalisation in Senegalese sole larvae at 30 dph: **e** control and **f** 1.56 μg/L malathion exposed larvae showing positive staining in thyrocytes and colloid content, and in plasma content from vasculature; **g** 3.12 and **h** 6.25 μg/L malathion exposed larvae showing a weak T_4_ immunostaining in the colloidal content but not in the thyrocytes, neither in the plasma content. Scalebar represents 300 μm

Morphologically, all controls showed a thyroid gland dominated by larger follicles lined by cuboidal or columnar cells depending on their secretory activity, that surrounded an inner colloidal lumen (Fig. 4a). During the larval development, the thyroid follicles increased in number and size up to 6–7 follicles that were visible at 30 dph (Table 1).

**Table 1:** Differences in number and size (μm) of thyroid follicles after malathion exposure

Number/Size of follicles (mean ± SEM)	13 dph		15 dph		30 dph	
Control	2	25,6 ± 3.2	3–4	26,39 ± 2.86	6–7	75,44 ± 8.45
1,56 μg/L	2	24,55 ± 3.4	2–3	22,44 ± 1.98	5–6	69,83 ± 6.25
3,12 μg/L	1	20,22 ± 2.4	2–3	18,29 ± 1.99	3–4	53,2 ± 3.23
6,25 μg/L	1	19,17 ± 1.9	2	21,24 ± 3.4	2–3	35,17 ± 1.9

In malathion exposed larvae, a significant reduction in both size and number of thyroid follicles was recorded in a dose-dependent way (Fig. 4 b to d). Thus, at 30 dph the size of the thyroid follicles ranged from 75,43 μm in controls (Fig. 4 a; Table 1) until 69.8, 53.2 and 35,2 μm in the different malathion treatments (Table 1), at 1.56 (Fig. 4) b, 3.12 (Fig. 4)c and 6.25 (Fig. 4)d μg/L, respectively. Additionally, the number of the thyroid follicles was also significantly reduced, ranging from 6 to 7 in control larvae up to 2–3 follicles in the exposures of 6.25 μg/L (Fig. 4 a to d; Table 1). At this higher concentration, epithelial cells of the thyroid follicles seemed to be atrophied, with signs of hyperchromatism (Fig. 4 d). Parallely to the reduction in size and number of follicles, a decreased functionality of the thyroidal follicles was detected by means of immunohistochemistry. Thus, whereas in controls at 30 dph, T_4_ immunostaining was evidenced in the thyrocytes, within colloid and in plasma contents, from the external irrigating blood vessels surrounding the thyroid follicles (Fig. 4 e and f), in malathion exposed larvae a weak immunostainning affinity for T_4_ was only present in the colloidal content. Additionally, thyrocytes or surrounding blood vessels were devoid of T_4_ immunostaining (Fig. 4 g and h). Similar results were recorded for T_3_ immuno-localisation (data not shown).

**Fig. 5:**
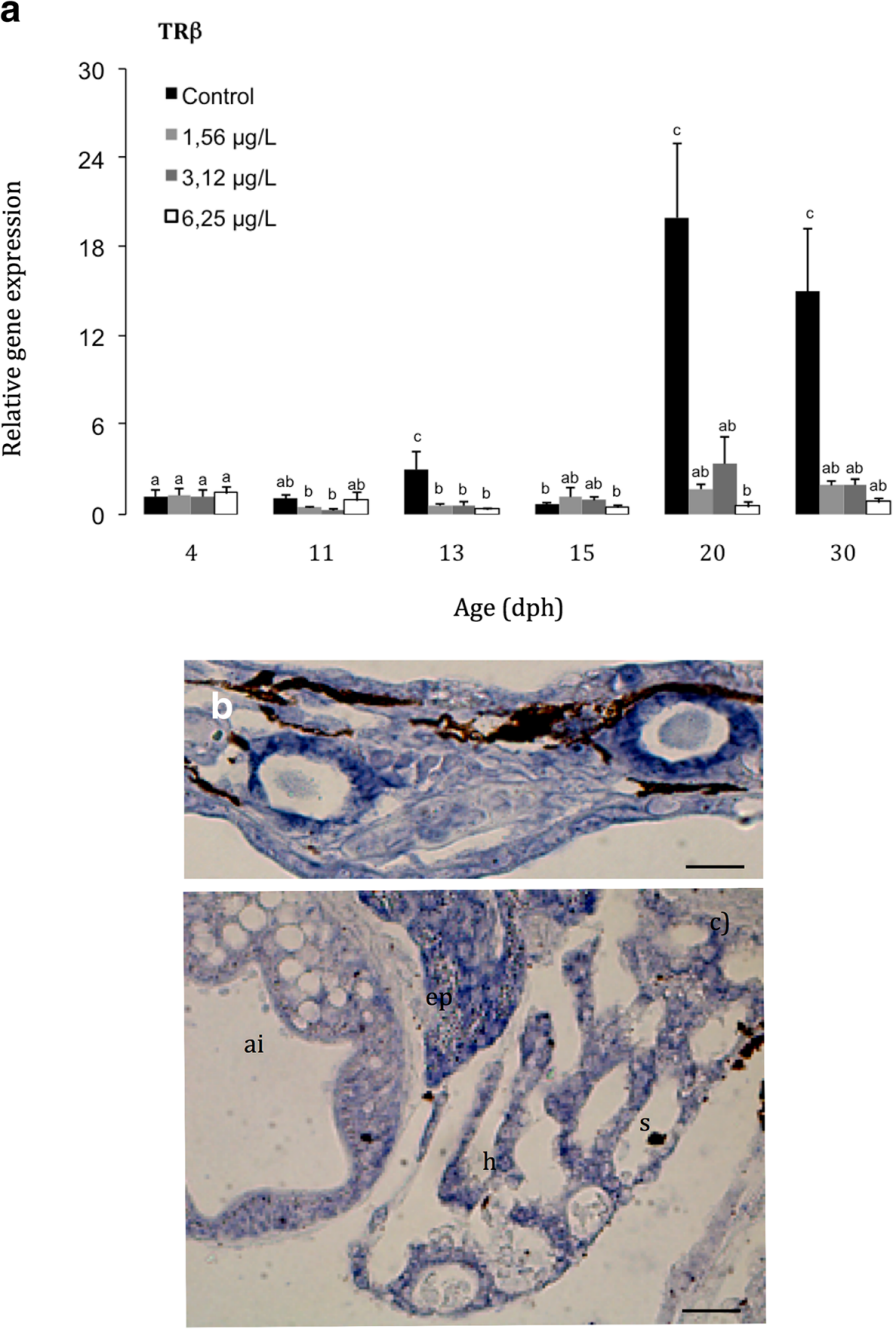
**a** Relative expression of the thyroid receptor at different developmental stages during the metamorphosis process of the Senegalese sole; Tissue detection of *Trβ* in thyroidal cells **b** and in different somatic tissues **c** by in situ hybridization. Scalebar represents 300 μm. Ai: anterior intestine; S: sinusoids; h: hepatocytes; ep: exocrine pancreas

The gene expression transcript levels of the thyroid receptor-*β* (*Trβ)*, it has been scarcely expressed before starting the metamorphosis (between 4 and 11 dph) in the controls. During the larval development, *Trβ* transcripts increased progressively from 13 dph onwards, displaying the highest *Trβ* expression levels at the end of metamorphosis stage, i.e. 18 fold increases of *Trβ* transcripts at 20 dph, in comparison with the pre-metamorphosis phase, and high levels of expression were also registered at the post-metamorphosis (at 30 dph). On the other hand, malathion exposed larvae exhibited between 42 and 19 fold lower *Tr*β mRNA levels (*p* < 0.05) than controls, at 20 and 30 dph, respectively (Fig. 5a). Furthermore, the *Tr*β transcripts were also detected by in situ hybridization in different cell-types of the *S. senegalensis*, in the thyroidal cells, as well as in several somatic organs-systems and tissues, eg., digestive, heart, skeletal and muscle structures (Fig. 5 b and c).

### Effects on skeletal development

**Fig. 6:**
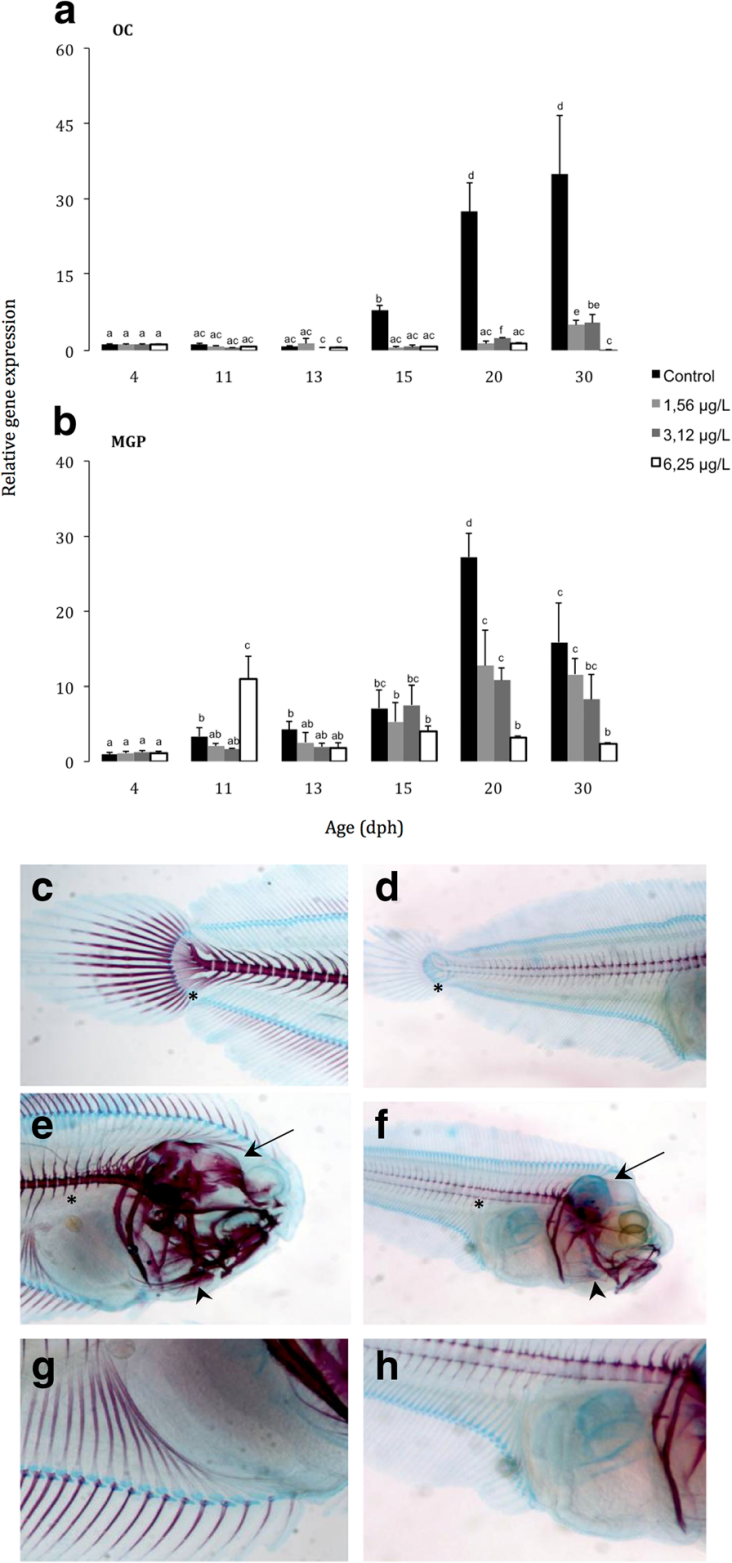
(**a** and **b**) Relative *Oc* and *Mgp* gene expression levels in Senegalese sole during larval development, in response to the different malathion treatments. Note in exposed speimens a strong down regulation of *Oc* expression from 15 dph onwards (**a**) and a moderate down regulation of *Mgp* expression levels from 13 dph onwards (**b**) when comparing with controls; **c** to **h** AA-AR staining in Senegalese sole at 30 dph showing for controls almost all calcified (red colour) structures **c**, b and **e** whereas for 6.25 μg/L malathion exposure, still remained blue in colour, indicating a cartilaginous composition (**f**, **g** and **h**)

The gene expression patterns of the OC and MGP and protein accumulation were analysed in both, the controls and malathion exposed fish. The progress of ossification and the changes induced in response to different malathion treatments were also studied by means of the Alcian blue and Alizarin red (AA/AR) staining. In controls, the expression levels of both *Oc* and *Mgp* mRNA transcripts were precociously registered during the metamorphosis (from 4 dph onwards) with lower transcript levels for *Mgp* than for *Oc,* until 15 dph, in which significant increased levels of both trancripts were evidenced. At the end of metamorphosis and postclimax-stages (at 20–30 dph), transcript mRNA levels slightly decreased for *Mgp,* however, the Oc trancripts remained at high levels of expression (Fig. 6 a and b). In malathion exposed specimens, a strong down-regulation of *Oc* mRNA transcript levels was detected from 15 dph onwards, in a concentration-dependent manner (Fig. 6a) for all assayed conentrations, whereas the mRNA Mgp transcripts displayed a significant peak of induction at 11 dph (3-fold increases) and a moderate although significant down regulation of *Mgp* expression levels from 13 dph onwards was recorded in comparison with the typical normal baseline expression patterns of the controls (Fig. 6b). Surprisingly, no appreciable differences in the tissue-localisation of *Oc* and *Mgp* transcripts were detected in cartilage and bone structures, from both controls and malathion exposed fish, as detected by in situ hybridization (data not shown).

The AA-AR staining revealed that sublethal exposure to malathion provoked a delay in bone development and ossification processes during development of the Senegalese sole (Fig. 6 c to h). In controls, at 30dph, an extended alizarin red staining was observed in several structures: lower and upper jaws, cranial bones, sphenoid, ceratobranchials, cleithrum, mesethmoid, lateral ethmoid, vertebral centra, neural and haemal spines (Fig. 6 c, e and g). However, in larvae exposed to the highest malathion concentration (at 6.25 μg/L) the alizarin red staining was restricted to neural and haemal spines, cleithrun, ethmoid, ceratobranchials, lower and upper jaws, while cranial bones or sphenoid, caudal fin complex, dorsal and ventral pherigophores and fin rays still remained stained in blue colour, indicating a cartilage composition with specific affinity to Alcian Blue (Fig. 6 d, f and h).

**Fig. 7:**
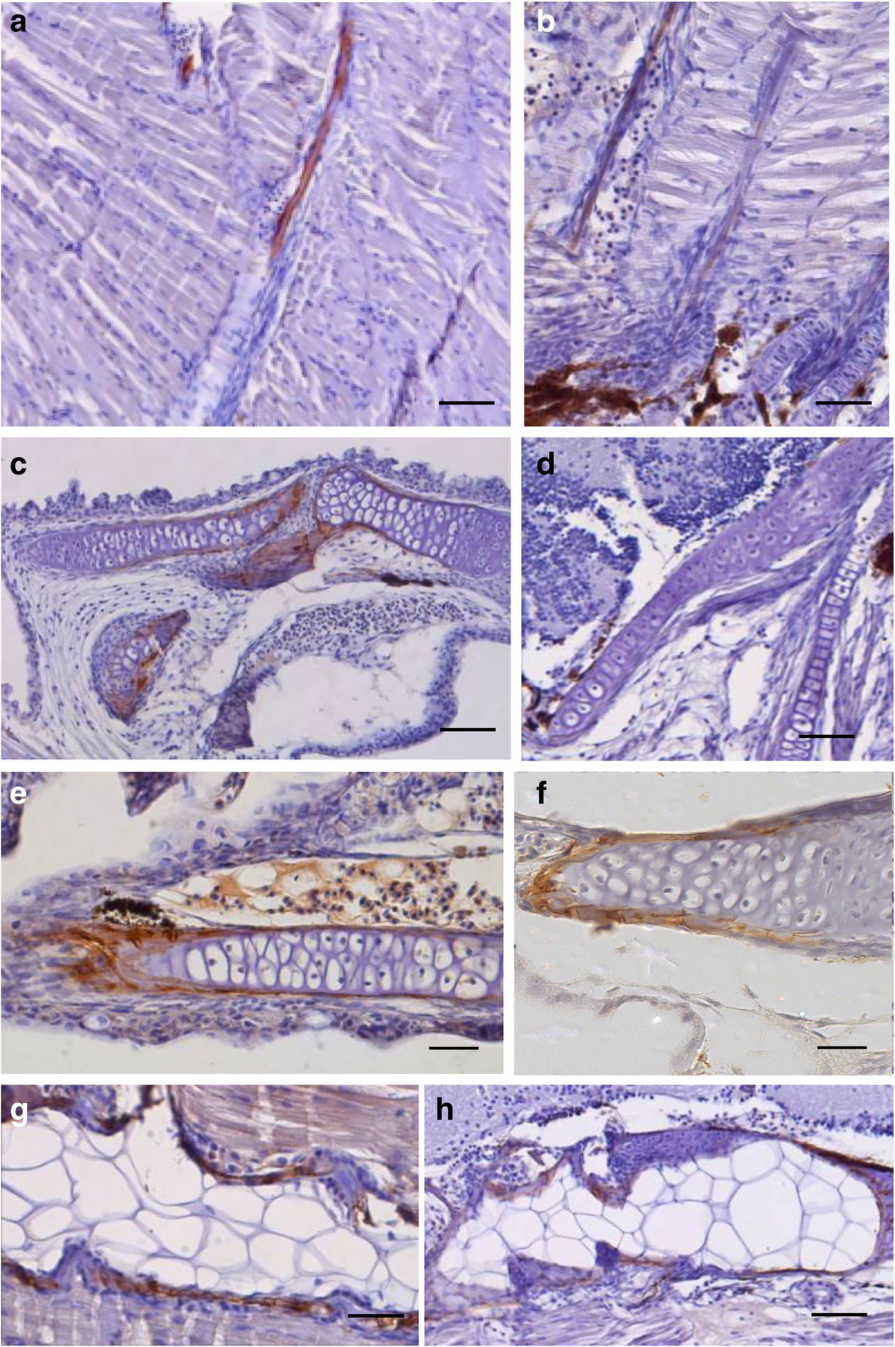
Immunohistochemical distribution of OC (**a** to **d**) and MGP (**e** to **h**) proteins in juvenile specimens of the Senegalese sole controls and malathion (6.25 μg/L) exposed specimens at 30 dph. Scalebar represents: **a**), **b**), **e**) to **h**) 300 μm; **c** and **d**), 100 μm

In the Fig. 7 is graphically represented the immunohistochemical distribution of the OC (Fig. 7 a to d) and MGP (Fig. 7 e to h) proteins, in juveniles of the senegalese sole, from controls and malathion exposed specimens at 30 dph. In the controls, the OC displayed a positive immunosignal mainly in the matrix of skeletal structures already calcified or under calcification, such as neural and haemal spines and in perichordal ossification zones from the head and bucoparhyngeal cavity, among others (Fig. 7 a and c). However, in exposed larvae (at 6.25 μg/L) no OC- immunostaining was registered (Fig. 7 b and d). The MGP was also accumulated in the controls, as for instance, in the mineralizing matrix of endochondral and intramembranous calcifyng bones such as vertebrae and cranial structures and in the calcified matrix below the trabecula. The MGP immunostaining was also located in the mineralizing branchial arches, and in non-mineralized walls (endothelia) of the vascular system (aorta and cardiac arterial bulbus) (Fig. 7 e and g). Interestingly, in exposed specimens (at 6.25 μg/L) MGP was located in the same areas as in the controls, but showing a weaker immunostaining intensity-pattern (Fig. 7 f and h).

### Eye development and disruptions

**Fig. 8:**
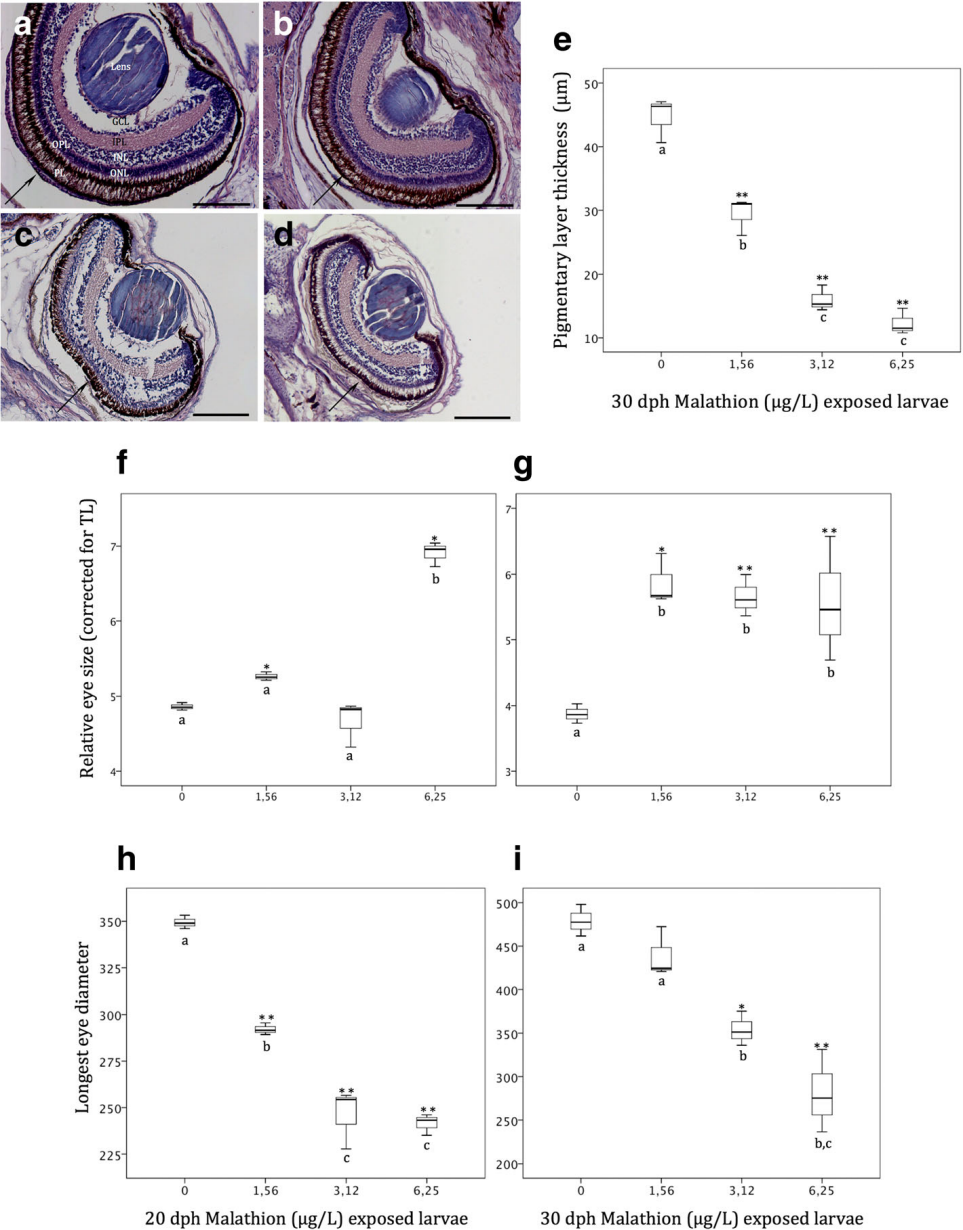
(**a** to **d**) Histological sections of the Senegalese sole larvae at 30 dph, showing the different retinal layers from controls (**a**), and from 1.56 (**b**), 3.12 (**c**) and 6.25 (**d**) μg/L malathion exposed organisms. Note reduction in size as well as in thickness of the pigmentary epithelium; **e** Changes in pigmentary layer thickness (μm) in response to the different treatments; Eye size corrected for total body length of larvae at 20 (**f**) and 30 (**g**) dph; Changes in the longest eye diameter at 20 (**h**) and 30 (**i**) dph in response to the different treatments. Different letters indicate statistically significative differences between treatments. (* = *P* < 0.05; ** = *P* < 0.01). Scalebar represents 100 μm

At the end of the experimental period (at 30dph), the larvae exposed to malathion displayed a reduction of the eye-size, and also a minor pigmentation pattern of the retinal pigmented layer (RPE) (Fig. 8a to d). In addition, the pigmentary epithelium displayed a significant reduction, of its thickness, in a concentration-dependent manner (Fig. 8e). Thus, in controls (30 dph), the eyes had a pigmentary epithelium of an average thickness of 43 μm, which significantly decreased to 12 μm, in those larvae exposed to the highest (6.25 μg/L) malathion concentration (Fig. 8e). Measurements of the lens and from the other retinal cell layers (ONL, OPL, INL, IPL, GCL) revealed no significant differences, between controls and treatments (data not shown). On the other hand, the relative eye-size corrected for total length (TL) significantly increased in those larvae exposed to the highest malathion concentration (at 6.25 μg/L), in larvae at 20 dph, and for those larvae exposed to all the concentrations assayed at 30dph (Fig. 8 f and g). Nevertheless, the eye-size expressed as the longest eye-diameter, gradually decreased as malathion concentration increased, being the eye diameter in malathion exposure at 6.25 μg/L, between 1,4 and 2-fold lower in comparison with the controls, at 20 and 30 dph, respectively (Fig. 8 h and i).

**Fig. 9:**
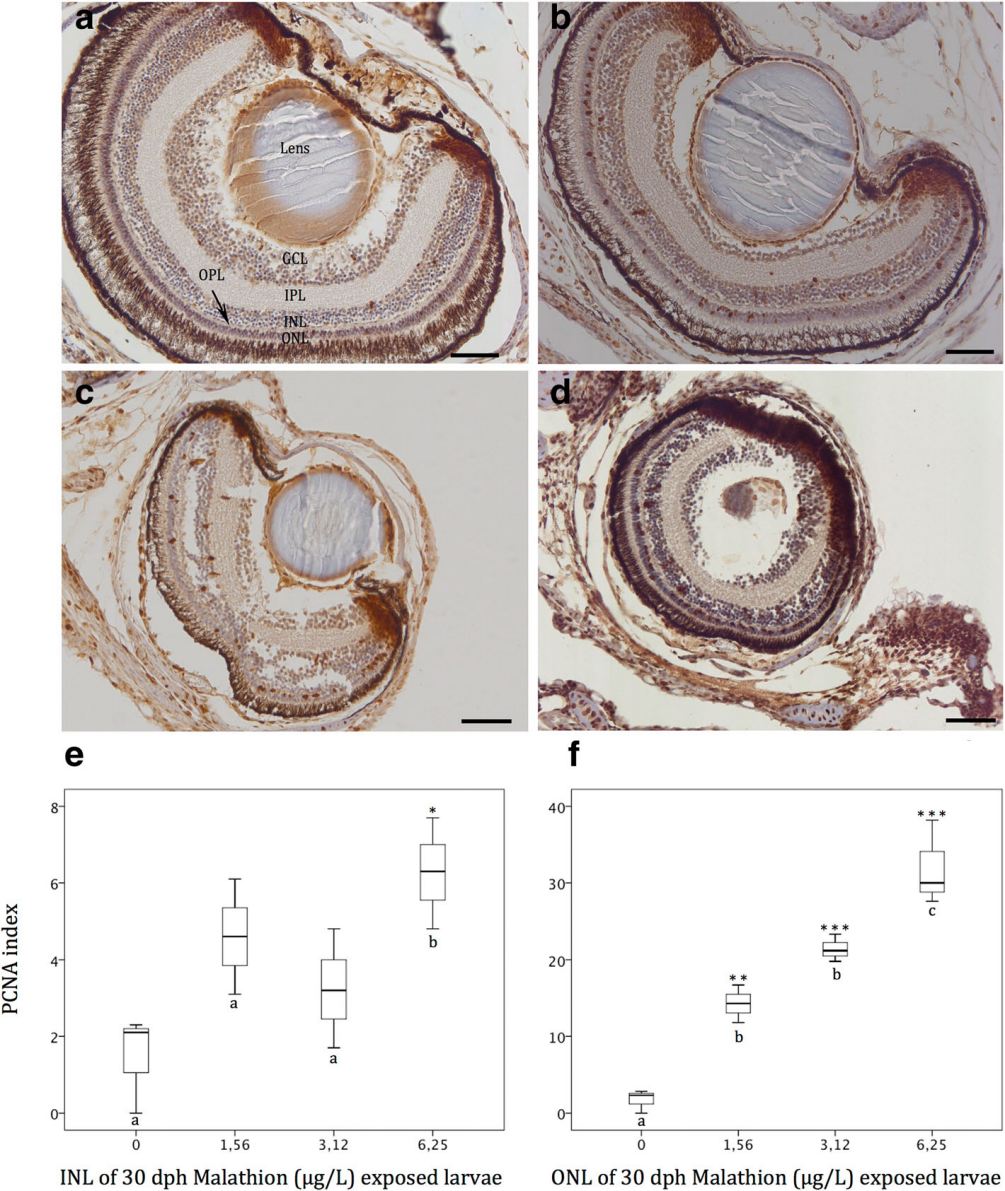
(**a** to **d**) PCNA immunolocalisation in different retinal layer from controls (**a**) and 1.56 (**b**), 3.12 (**c**) and 6.25 (**d**) malathion exposed organisms. **e** and **f** PCNA index calculated in the inner and outher nuclear layers respectively at 30 dph of malathion exposure. Note a significant increase of cell proliferation as malathion concentration increased in both inner (**e**) and outher (**f**) nuclear layers at 30 dph. Different letters indicate statistically significative differences between treatments. (* = *P* < 0.05; ** = *P* < 0.01; *** = *P* < 0.001). Scalebar represents 100 μm

The immunohistochemical detection of proliferative cells, by using PCNA (i.e. proliferating-cell nuclear antigen) antibody revealed cell proliferation patterns in both, the retinal INL and in the ONL layers in malathion exposed fish, at 30 dph (Fig. 9 a to d) when compaing to controls. Cell proliferation quantified as PCNA index significantly increased in both, the inner and the outer nuclear retinal layers, in the larvae of the Senegalese sole exposed to malathion in a dose-dependent manner (Fig. 9 e and f), during the first month of life.

## Discussion

Many environmental chemical contaminants, phytochemicals, and other natural and synthetic xenobiotics, that are known as typical endocrine-disrupting chemicals (EDCs), or acting as selective estrogen receptor modulators (SERMs), can affect wildlife and humans by interfering with endocrine homeostasis and hormonal processes, e.g. oestrogen or thyroid signaling acting through the hypothalamus-pituitary-thyroid (HPT) axis [16, 17, 22], among others. Indeed, many of the EDCs act interfering with the thyroid function and/or blocking different signals of the thyroid cascade, i.e., hormones, receptors, enzymes, transporters, etc., [9, 31, 56]. Accordingly, in this context, the organosphosphate pesticide (OP) malathion, that is a specific inhibitor of the enzyme acethylcholinesterase (AchE), may act interfering with sensory receptors, i.e. chemoreception, especially olfaction and vision [15]. In addition, in fish species many behaviors are affected by the disruption of the neuro-endocrine system, because it is very sensitive to different contaminants, enclosing OP pesticides, that are also known acting as typical endocrine-thyroid (and oestrogenic)-disruptors ([9, 57, 58]. The OP malathion has been reported to disrupt the thyroidal homeostasis, and to compromise fish growth, behavior and fitness, as well as to alter the larval development, cell-tissue differentiation, metamorphosis, reproduction, and healthy survival [2, 4, 9, 13, 14, 55]. On the other hand, in vertebrates, but most particularly in anuran amphibians, as well as in fish, especially in Pleuronectiform flatfish species, such as the Senegalese sole*,* the metamorphic process by which a symmetrical larva becomes an asymmetric juvenile, is a thyroid-driven process, although many other endocrine signals and different biotic and abiotic factors also influence this complex-process [9, 13, 21–23, 35]. Interestingly, the flatfish metamorphosis is accompanied by an extensive craniofacial remodeling and migration of one eye, among other ontogenetic changes and biological, behavioral and ecological adaptations [22, 23]. In flatfish species, the most common developmental abnormalities include lack of eye migration, bone deformities, and pigmentation disorders, which have been related with zooctechnical procedures, genetic and nutritional factors, and environmental contaminants [14, 22, 33, 59, 60], among others.

### Effects of malathion on growth patterns

Ecotoxicological purposes often evaluate variations of growth patterns, because it reflects different molecular and cellular responses, as one easily measurable biometric parameter [61, 62]. Considerable number of reports, on retardation and decreases in fish growth, following exposure to pesticides and other chemicals are available [63–67]. Particularly, the OP pesticide malathion induced shortening of body length, as well as circulatory and collagen defects in different fish species, enclosed early lifes stages of the Senegalese sole [55, 68, 69]. In zebrafish embryos, the exposure to malathion (at 3 mg/L), reduced the body-lengths up to 80% with respect to the controls [63]. In the catfish, *Clarias batrachus*, exposed to malathion (0.001 mg/L), Lal [2] reported a significant decrease in the body and ovarian weights, during different phases of the reproductive cycle. Similarly, in the present study, the exposure to malathion affected the Senegalese sole growth patterns, in terms of total length (TL) and dry weigh (DW) in a dose dependent-way. In fact, the exposure to the highest malathion concentrations (at 6.25 μg/L) reduced significantly the length and weight (up to 60 and 92%) with respect to the controls, respectively. Among malathion’s mechanisms implicated in reduction of fish growth, and in the ontogenetic developmental alterations, several dependent thyroidal disrupting effects have been pointed out. It has been suggested that malathion not only arrests the T_4_ secretion, but it also inhibits the conversion of T_4_ into T_3_. Besides, it has been reported that T_3,_increased the growth patterns in the yearling coho salmon, whereas T_4_ enhanced food consumption [70, 71]. In *Clarias batrachus* exposed to malathion, Lal [2] reported a drastic reduction in the body-weight, as well as in the food intake due to a disruption of the endocrine machinery associated with the regulation of food-consumption and its metabolism, what provoked a growth retardation. In addition, the exposure to malathion disrupted the endocrine funntioning, and the olfactory sensation responsible for food uptaking, and gustatory feeding behavior, which ultimately leads to retardation of the fish growth [7].

On the other hand, in flatfish species, as the Senegalese sole, the progression of the metamorphosis is analysed by the position of the left-eye, and well evaluated by means of the eye migration index (*I*_EM_) [34, 72]. In the present study, this *I*_EM_ showed significant differences after exposure to malathion in a dose-dependent manner. In this context, *I*_EM_ was delayed from 13 dph onwards, in those larvae exposed to malathion (at 1.56 and 3.12 μg/L), and from 12 dph onwards, with the highest concentration of malathion (at 6.25 μg/L). At the end of the experimental period (at 30 dph), larvae exposed to 1.56 and 3.12 μg/L presented *I*_EM_ values of 3 and 2.12 respectively, whereas in those larvae exposed to the highest concentration of malathion (at 6.25 μg/L) showed the lowest values of *I*_EM_ = 0.44. According to previous reports [34, 72], in the present study, exclusively the controls completed the eye migration at 30 dph (*I*_EM_: 5). In different flatfish species, variable changes of the *I*_EM_ have been recorded in response to different stress conditions and nutritional and environmental factors. In this context, Hamre et al. [73], in the metamorphosis of the halibut indicated that the *I*_EM_ was affected by the composition of consumed preys. Sæle et al. [74] in the Senegalese sole, also observed differences in the eye migration index that depended on the quality of preys. On the other hand, Soffientino et al. [75] in larvae of the summer flounder exposed to contaminants (e.g., PCB-126) registered a delay of the metamorphic progress determined by a lower value of the *I*_EM_ and smaller fish size. Accordingly, in early lifestages of the Senegalese sole, a reduction of the growth patterns which is induced by the mañathion is clearly accompanied by a lower eye migration index and consequently with a delayed process of metamorphosis.

### Thyroid disorders

Thyroid hormonal disruptions induced by different xenobiotics, enclosing OP-pesticides, can influence directly the progress of metamorphosis [9, 13]. In different fish species, it has been suggested that the inhibitory effect of OP insecticides, as the malathion, among other xenobiotics acting as endocrine thyroid disruptors, could be attributed to changes and alterations in developing thyroid follicles, deficient iodide trapping, and/or inhibition of the enzymes necessaries for synthesis of T_4_, and thus conversion of T_4_ to T_3_ hormones [9, 76–78]. In comparison with the normal thyroid gland ontogeny and thyroidal hormonal patterns previously described on this flatfish species [31, 79, 80], among others, different ontogenetic and physiological damages, and thyroid disruptions were registered in malathion exposed *S. senegalensis* larvae, during metamorphic and post-metamorphic stages (at 13 and 30 dph): minor size and number of follicles, decreases in thyroid hormone production, detected by means of immunohistochemical procedures, and lower *Trβ* transcript expression levels. In parallel to these thyroid disruptions, significant delays or failures in the metamorphosis process, abnormal flatfish phenotypes, alterations and failure of eye migration (decreased *I*_EM_), as well as pigmentation and skeletal abnormalities have been registered in malathion exposed fish, and specially at the highest malathion concentration tested (at 6.25 μg/L), during the metamorphosis process (13–30 dph).

As previously described, the size and height of the follicular epithelium of thyrocytes is a marker of the secretory activity of the thyroid gland [79, 81]. The thyroid gland from controls is characterized by numerous and big follicles lined by cuboidal or columnar cells and can be classified as actively secreting gland whereas such as has been recorded in malathion exposed Senegalese sole specimens, those thyroid glands with scarce follicles lined by flattened epithelial cells, are classified as less active glands according to these last authors. These results were corroborated by immunohistochemistry in the controls, in which T_3_ and T_4_ immunostainings were strongly detected in cuboidal cells and colloid content from thyroid follicles, and in plasma content from vessels surrounding the follicles, indicating that the thyroid gland actively synthesize, accumulate and excrete hormones. Unlike, in malathion exposed fish, T_3_ and T_4_ immunoreactivities were scarcely and weakly located in the colloid content, but not in plasma, neither in follicle-surrounding epithelial cells. Similar results were reported in the catfish *Heteropneus fossilis* [82], and in *Clarias batrachus* [54, 83], in which the OP pesticide treatments induced a decline in thyroid hormone levels. In this context, Sinha et al. [83] reported that exposure to malathion appeared to decline the plasma T_3_ levels and T_3_/T_4_ ratio, suggesting that this pesticide not only blocks the T_4_ secretion, but also inhibits the thyroidal conversion of T_4_ into T_3_, by the action of deiodinases 1,2,3 [23, 31]. Furthermore, at the cellular level in several target organs, the thyroid hormonal signalling is generally initiated by the interaction of the active hormone, T_3_, and binding to the thyroid receptors (TRs). *Trα* is important for post-natal development, cardiac function, and regulation of deiodinases in cerebral cortex, while the *Trβ* mediates the regulation of the liver metabolism and thyroid hormones by a negative feedback through the HPT axis [22, 23]. Moreover, transcripts of *Trβ* are mainly expressed in the pituitary, liver and kidney, while *Trα* should be ubiquitously expressed (Kim et al. 2015). In different flatfish species, there is a concomitant rise of both hormone levels (T_3_, T_4_) and thyroid receptors (*Trα*, *Trβ*) during metamorphosis climax, and a subsequent decline toward metamorphic post-climax [22, 35]. Similar results were previously described in *S senegalensis* development, with parallel expression patterns for both *Trβ* and T_4_ levels. Moreover, it was pointedout that *Trβ* transcription is up-regulated by THs that play a major role during the metamorphosis [31, 80]. In the present study, the highest *Trβ* expression levels were reached at 13–20 dph and 20–30 dph (metamorphosis and post-climax), such as it has been previously reported on this flatfish [23, 31, 80]. A significant down-regulation of the *Trβ* expression levels after malathion exposure was recorded from 13 dph onwards coinciding with the onset of the metamorphosis phase (from 11 dph onwards). Decreased *Trβ* transcript levels in malathion-exposed larvae occurred in parallel with a decrease of the content of THs, such as it has been detected by immunohistochemistry, in the present study. It has been pointed out that the malathion modulates thyroid receptors-mediated gene expression, not only directly by interacting with the TRs, but also by controlling plasmatic T_3_ levels [77].

### Skeletal alterations

The baseline expression patterns of both OC and MGP, used as markers of bone and cartilage development respectively, as well as the immunohistochemical distribution of both proteins during the development and metamorphosis of the Senegalese sole, was previously reported by Gavaia et al. [36]. In the present study, the controls displayed low levels of the *Oc* mRNA transcripts, at early life stages (from 4 to 15 dph), and a significant up-regulation (increased *Oc* levels) was recorded from 15 dph until the end of metamorphosis. Unlike, in malathion exposed metamorphosing and post-metamorphosed larvae and post-larvae, a strong reduction in the expression levels of the *Oc* mRNA transcripts was registered. These results were sustained by immunohistochemistry data, in which the osteocalcin protein-OC was located in skeletal tissues underwent calcification in the controls, whereas it was devoid of OC-immunoreactivity, in the malathion exposed fish. Because the osteoblasts express acetylcholinesterase (AChE), any OP-pesticide that target and disrupt cholinergic signaling pathways, as the malathion, may affect bone development and remodeling via their effects on osteoblasts by reducing its cholinesterase activity [84–86].

In the present study, malathion exposed larvae showed a precocious peak of expression of the *Mgp* mRNA at 11 dph, that was restored to similar baseline levels that the controls at 13 dph, followed by a strong reduction of the *Mgp* transcript levels from 13 dph onwards, in comparison with controls, until the end of the experiment. Similar results were obseved by immunohistochemistry, since malathion exposed larvae showed a visible reduction of the MGP immunostained areas. Considering the role of the MGP, as a physiological inhibitor of calcification, the effects of the precocious expression of the *Mgp* mRNA on bone development and mineralization could be synergistic to the parallel decrease of the expression levels of the *Oc* mRNA transcripts, in those malathion exposed specimens, conducting to a delay in skeletal development. Because the MGP is secreted by chondrocytes and vascular smooth- muscle cells (vsmc), any compound that disrupt activity of these cells, like the malathion, may affect levels of MGP proteins, and therefore it will affect to bone development [85].

On the other hand, it is also assumed that, in fish species, the normal bone development can be disrupted by deficiences or by increases of thyroid hormonal levels, depending on stages of larval ontogeny and timing of transition into juveniles [22, 87]. In this sense, and for corroborating the influence of the thyroidal signaling pathways in the skeletal development, a close relation-ship between serum concentration of OC and thyroid status has been reported [88–90]. Another evidence concerning the role of thyroid in bone development is the fact that TRs which recognize specific response-elements in the promoter region of target genes, are present in the osteoblasts [91]. Therefore, this fact regulates the expression of non-collagenous proteins, like the osteocalcin-OC. Additionally, in a study regarding the role of thyroid hormones in tissue calcification, Sato et al. [92] concluded that the presence of T_3_ (3′,3,5-triiodo-L-thyronine) regulated the *Mgp* gene expression patterns in vascular smooth muscle cells. Furthermore, it has been reported that thyroid hormones regulate the expression of several genes implicated in bone formation, such as collagenase-3, gelatinase-3 and osteocalcin in osteoblasts-like cells culture [93].

In addition, it can be suggested that the OP malathion affects bone development and ossification processes by a direct disruption of the target cells (osteoblasts and mesenchymal stem derived cells-MSCs) that secrete these Gla-containing proteins (OC and MGP) or indirectly by affecting the thyroidal signaling pathways that regulates the expression of both the *Oc* or *Mgp* genes. In any case, both mechanisms can conduce to abnormal phenotypes, since significant delays in bone development and ossification processes were evidenced, showing many cartilage structures in advanced stages of larval development (at 6.25 μg malathion/L, at 30 dph), when bone structures should be ossified. There are numerous reports that support the role of the ossification index (performed by means of AA/AR technique) as a good biomarker of skeletal development and malformations [94–98]. In the present study, when skeletal phenotypes are analysed, it can be observed a different degree of mineralization, when comparing controls and malathion exposed specimens, in particular in certain structures from those specimens exposed to the highest malathion concentrations (at 6.25 μg/L). In fact, in controls at 30 dph, it was recorded an extended alizarin- red staining located in lower and upper jaws, cranial bones, sphenoid, ceratobranchials, cleithrum, mesethmoid, lateral ethmoid, vertebral centra, neural and haemal spines, whereas in the malathion exposed fish, cranial bones, dorsal and ventral pherigophores, fin rays or sphenoid were devoid of AR staining, suggesting that skeletogenesis was arrested, by effect of the OP malathion.

### Eye development and disruptions

Eye development in the flatfish, *Solea senegalensis* is a remarkably fast process that gives to fish, the visual capabilities for prey capture, and feed needs, at the early larval phase of the ontogeny [37], such as it has been reported in many other fish species, and in all vertebrates [22, 23]. Thyroid system is a crucial key for eye development in vertebrates, and therefore, any disruption in the thyroid axis might affect the eye morphology of developing flatfish species [23]. As a consequence, fish larvae exposed to thyroid disruptors, as the OP malathion, become particularly vulnerable, because vision and other sensory organs are impaired particularly during the metamorphosis process [99]. In the present study, at the beginning of the metamorphosis in the controls, both eyes are well developed, differentiated, and under maturation processes, with all structurally formed retinal layers and cell types (pigment epithelium, nuclears, plexiforms, ganglion cell layers among others), and showing different functional retinal neuromarkers, such as it was previously reported on this flatfish species [37, 100]. On the other hand, malathion exposed specimens displayed, at 30 dph, a decreased developmental pattern, in both the eye size and thickness of the retinal pigment epithelium (RPE). Thus, the eye-size in terms of longest eye diameter was reduced from an average of 0,48 mm for controls, to 0,24 mm for 6.25 μg/L malathion exposed fish. Similar results were reported by Cook et al. [63] in juveniles of the zebrafish exposed to malathion, in which a significantly reduction of the eye-size was detected. However, the relative eye-size corrected for total length significantly increased in a dose-dependent way, at 20 and 30 dph, suggesting that the observed reduction in eye-size likely could be an overall developmental delay of the different organs-systems, rather than an specific and particular effect on the eye development provoked by the malathion [101]. In the present study, and parallelly to a decrease of the eye-size, in malathion exposed specimens, an increase of the PCNA index, in both the ONL and INL retinal layers, in a dose-dependent manner, was detected. As suggested by Negishi et al. [102] in a study in goldfish treated with neurotoxins, a cell damage in the retinal cells induced by toxins, provoked increases in the number of PCNA-positive cells, which could indicate a cell over-production or increased cell-proliferation rates for repairing of retinal toxicant injury.

In the Senegalese sole, parallely to eye development and differentiation, the migration of the left eye occurs during the metamorphosis. Several variants of the eye migration and adaptive behaviors in response to malathion treatments have been detected in the present study at 30 dph: (1).- Bilateral symmetry with upright swimming and planktonic behaviors in the majority of the sampled fish (up to 75%) exposed to 6.25 μg/L; (2).- Bilaterally symmetric eyes with lateralized behaviors with their left sides on the bottom in half of Senegalese sole larvae exposed to 3.12 μg/L (up to 40%) and in some of the larva exposed to 1.56 μg/L (up to 15%) and (3).- Incomplete eye migration with one of the eyes partially repositioned to the other side in some of the exposed to 1.56 μg/L (up to 35%) and finally (4).- Complete eye migration such as it has been observed in all sampled controls and in a half of the fish exposed to 1.56 μg/L (up to 50%). Although it was previously pointed out that lateralized behaviours are adaptative responses for changing the eye position [103], Schreiber [104] in other study in southern flounder (*Paralichthys lethostigma*), in pre-metamorphic larvae treated with thyroid hormones concluded that although lateralized behaviours and eye migration occurred, in response to thyroid hormones during metamorphosis, both processes are independent to each other [104]. Further, these authors suggested that the development of lateralized behaviors was a result of a vestibular response to thyroid hormones rather than an adaptive response to eye translocation. In this sense, morphological asymmetries of the otoliths as well as of the utricles and saccules from the inner ear, have been detected in adult flatfish [105–107]. In flatfish species, the process of eye migration starts with an asymmetrical growth of neurocraneal bones, the frontal bones and paraethmoids, initially located in symmetrical position between the eyes. The movement of this bone structure exerts a stretch on the fibroblast in the connective tissues found between the moving structures and one eye migrates to the other side of the body [108]. However, there could occur a physical blockage of eye extension if that asymmetrical ossification of cranial bone structures is disrupted [109]. Because bone ossification and remodeling are thyroid-driven processes, exposure to any contaminant that can affect the thyroid axis can also alter bone development and, consequently, the appropriate eye migration [110], such as was detected in Senegalese sole exposed to the OP-malathion (6.25 μg/l, at 30 dph).

## Conclusion

In conclusion this study confirms that the subacute exposure to environmentally relevant sublethal concentrations of the OP malathion particularly during the Senegalese sole metamorphosis significantly altered the ontogenetic developmental events and growth patterns, by a direct toxic effect over target cells, organs and tissues (i.e. thyroid, eye, skeleton). Additionally, all of the induced disruptions may affect to the functionality of these organs and tissues, and/or indirectly altering the thyroid signaling cascade, as for instance, to block hormonal synthesis, secretions, and thyroidal homeostasis. Accordingly, all these potential umbalances may lead to noticeable metamorphic failures, through several disruptive thyroidal processes, which may arrest the onsent and advancement of the most important ontogenetic metamorphosing events, such as the migration of the eye, skeletal development and bone remodeling, and the acquisition of benthic life and settling behaviors.

## Methods

### Larval rearing and experimental assays

Newly hatched *S. senegalensis* larvae (provided by the IFAPA-El Toruño; Regional Government of Andalusia, Spain) were reared at the ICMAN-CSIC (Puerto Real, Cadiz, Spain). Larvae (*n* = 60 per L) were distributed into fifteen 16 L cylindro-conical tanks (control+control/carrier+ 3 malation concentrations x triplicate), and used for different experimental assays. Water temperature was maintained at 20 ± 0.4 °C, salinity 32–38 gL^− 1^, and dissolved oxygen 85–100%. Photoperiod was maintained at 12 h light: 12 h dark and mild aireation was provided. Larvae were fed rotifers from 3 to 9 days post hatching (dph) at a density of 15 organisms per ml and artemia nauplii from 6 dph at a initial density of 1 organism per ml. Artemia density was gradually increased, becoming the only prey added from 5 dph onwards, according to previous studies [79].

Recent results performed in *S. senegalensis* larvae exposed to malathion revealed that the toxic-sublethal concentrations of this OP insecticide ranged between 1.56 and 6.25 μgL^− 1^ [55]. Larvae from 4 dph and during the first month of life (on day 30) were exposed to malathion (Sigma Aldrich Chemical Co, St Louis) under semistatic-renewal toxicity tests (50% of renewal each 24 h). Three different nominal concentrations of malathion (1.56, 3.12, 6.25 μgL^− 1^) were tested. All experimental treatments and control received the same carrier solvent (0.01% acetone, maximum acceptable limit for solvent with no observable effect, according to OECD guidelines [111, 112]. A control group without solvent was also handled in parallel with the experimental assay. Three replicates were performed for each test (control and treatments).

All experimental designs and fish handling were performed in accordance with the European Directive and followed the Spanish normative (RD 1201/2005) and procedures of animal welfare (ICMAN-CSIC facilities; REGA-ES110280000311). The experimental procedure (Proyect AGL2010–15951) was approved by the Spanish National Research Council (CSIC) Ethical Committee, and Ethical Committee for Animal Welfare of ICMAN-CSIC.

All experiments were performed with Senegalese sole larvae from 4 dph onwards, in order to avoid the high natural mortality, that is very common, during the endogenous feeding phase of this flatfish species.

### Biometric parameters

Standard length (SL) and dry weight (DW) were measured in larvae at 5, 10, 20 and 30 dph. Thirty larvae from each experimental tank were randomly sampled and euthanized with an overdose of tricaine methane sulphonate (MS-222, Sigma Aldrich). SL was measured using a stereomicroscope equipped with an eye-piece with a metric scale. DW calculation was performed according to Fernández-Díaz et al. [34] by rinsing larvae with distilled water to remove salt and then oven-drying at 60 °C for 24 h. Samples were weighed with an analytic microbalance.

The eye migration index (*I*_EM_ = Σ(% fish in each stage x stage)/100) is ussually used in the Senegalese sole development, as a valuable measurement tool for evaluating the metamorphosis advancement. In our experiment, eye migration was assessed at 6, 12, 13, 15, 20 and 30 dph (15 larvae per replicate) according to Villalta et al. [72]. Data are presented as the relative number (percentage) of larvae at each stage of development, i.e. at S0, S1, S2, S3a, S3b, S4 (to see [113]), and evaluated at the same sampling time.

### Histological procedures

For histological purposes, a RNAse free procedure was used according to previous studies [114]. At the end of the experimental period (on day 30), about ten to fifteen larvae per each treatment (3 independent replicates, respectively) were sampled and fixed with 4% freshly prepared paraformaldehyde (PFA) in diethylpyrocarbonate-(DEPC)-treated phosphate buffered saline for 24 h. Afterwards, samples were stored in methanol at − 20 °C and then processed according to Ortiz-Delgado et al. [79, 114] Serial sagittal and coronal sections (5–6 μM thick) were used for the different histological approaches.

### Eye development

Serial coronal sections (=5 μM) from both control and exposed larvae were stained with Haematoxylin-Eosin (HE), and analysed using a light microscope (BX41 Olympus light microscope equipped with an Olympus digital camera (c3030)). Eye-size was measured as the longest diameter of the eye. Relative eye-length, was calculated as the ratio of eye length over total length expressed as apercentage. Differences in thickness of the different retinal layers (PL: pigmentary layer; ONL: outer nuclear layer; OPL: outer plexiform layer; INL: inner nuclear layer; IPL: inner plexiform layer; GCL: ganglion cell layer) in response to the OP malathion treatments were also analysed.

To measure cell proliferation index, in the different retinal layers, immunohistochemical approaches were assessed by using as primary antibody, anti-proliferating cell nuclear antigen/anti-PCNA (Santa Cruz Biotechnology) such as was described by Bakke-McKellep et al. [115]. Quantification of cell proliferation was conducted according to Sirri et al. [116]. From each histological section, several microphotographs were performed from various retinal areas, with different magnifications. Strongly PCNA-immunoreactive (PCNA-ir) cells were counted per area (0.1 × 10^4^ μm^2^), in the outer and inner nuclear layers (ONL and INL respectively) except for retinal margins, where PCNA-ir cells were densely packed. Data from different retinal regions were used to calculate PCNA index according to Lee et al. [117] as follows:

$$ \mathrm{PCNA}\kern0.5em \mathrm{index}=\frac{\mathrm{Number}\kern0.5em \mathrm{of}\kern0.5em \mathrm{PCNA}-\mathrm{positive}\kern0.5em \mathrm{nuclei}}{\mathrm{Number}\kern0.5em \mathrm{of}\kern0.5em \mathrm{PCNA}-\mathrm{positive}+\mathrm{negative}\kern0.5em \mathrm{nuclei}}\times 100. $$

### Thyroid gland development

The development of the thyroid gland (number and size of thyroidal follicles) and the immunohistochemical detection of THs, thyroxin (T_4_) and triiodothyronine (T_3_) were evaluated on histological sections of larvae and post-larvae at 13, 15 and 30 dph (*n* = 3 specimens per experimental condition x triplicate: *n* = 9) according to Ortiz-Delgado et al. [79] by using monoclonal antibodies (Cat. 10-T35A M94210–515 and Cat. 10-T30B M94207–3010 from Fitzgerald (USA), respectively) previously validated for this species.

### Skeletal staining analysis

To evaluate the impact of different malathion concentrations on the ossification of the skeleton, 20 larvae per each experimental tank were randomly sampled at the end of experimental period (at 30 days), fixed in 4% formaldehyde at pH = 7.0 with 0.1 M phosphate buffer. Fish were stained with Alcian Blue (pH:2.5) and Alizarin Red to detect cartilaginous and bony tissues, respectively [118]. Skeletal structures were identified according to Gavaia et al. [118] and Wagemans and Vandewalle [119].

The differences in the distribution patterns of osteocalcin-OC (as a marker of calcified bone tissue) and matrix Gla protein-MGP (a calcification inhibitor of soft tissues), as response to malathion treatments, were determined by immunohistochemical approaches [120]. Briefly, serial histological sections were incubated overnight with primary antibodies at suitable dilutions (1500 for OC, 1:250 for MGP). After incubation, sections were washed 3 times for 30 min each in PTW and incubated for 1 h with secondary antibody (goat anti rabbit IgG peroxidase conjugated at 1:1000 (Sigma, St Louis, MO, USA)). Peroxidase immunodetection was achieved by staining with 3,3′-diaminobenzidine/DAB as chromogenic substrate, in Tris-Hcl 0.05 M, at pH: 7.6 containing 0.05% hydrogen peroxide. To confirm the specificity of antibodies, negative controls were performed by replacing the primary antibody with pre-immune serum or BSA and by omission of primary or secondary antibodies.

### RNA isolation and cDNA synthesis

Pools of larvae of different ages (from 4, 11, 13, 15, 20 and 30 dph) were collected, washed with DEPC water, frozen in liquid nitrogen and stored at − 80 °C until RNA extraction. Total RNA was isolated from 40 mg of pooled larvae using NucleoSpin®RNA II kit (Macherey-Nagel, Düren, Germany) coupling with Lysing Matrix D (Q-BioGene) for 40s at speed setting 6 in the Fastprep FG120 instrument (Bio101), according to the manufacturer’s instructions. In all cases, total RNA was treated twice with DNase I using the RNase-Free DNase kit (Qiagen) for 30 min in order to avoid amplification of contaminated genomic DNA. RNA sample quality was checked using Experion (Bio-Rad) and quantification was performed spectrophotometrically. Total RNA (1 μg) from each sample was reverse-transcribed using the iScript™ cDNA Synthesis kit (Bio-Rad). Lack of genomic DNA contamination was confirmed by PCR amplification of RNA samples in the absence of cDNA synthesis.

### Real-time q-PCR

Real-time analysis was carried out on the iCycler (Bio-Rad). Reactions were accomplished in a 25 μl volume containing cDNA generated from 10 ng of original RNA template, 400 nM each of specific forward and reverse primers, and 5 μl of SensiFAST SYBR No-ROX (Bioline). Matching oligonucleotide primers were designed for *Oc*and*Mgp* genes using the Oligo v6.89 software (Medprobe) and sequence database GeneBank or SoleaDB (Table 2). To *Trß* gene, primers SseTrßF3 y SseTRrß4 were used [80]. Amplification of cDNA fragments encoding target genes was verified in previous assays by direct sequencing of PCR products obtained with the same reaction conditions employed in real-time PCR. The real-time amplification protocol used was as follows: initial 7 min denaturation and enzyme activation at 95 °C, 40 cycles of 95 °C for 15 s, and 68 °C for 30 s. Each assay was done in duplicate. For normalization of cDNA loading, all samples were run in parallel using ubiquitin as housekeeping gene with primers SseUB1 and SseUB2 (Table 2) according to Infante et al. [121]. To estimate efficiencies, a standard curve was generated for each primer pair based on known quantities of cDNA (10-fold serial dilutions corresponding to cDNA transcribed from 100 to 0.01 ng of total RNA). All calibration curves exhibited correlation coefficients higher than 0.98, and the corresponding real-time PCR efficiencies were around 2.1–2.2 for both all genes. Relative mRNA expression was determined using the 2^−(ΔΔCt)^ method [122]. Results were expressed as mean ± SEM. Statistical analyses were carried out using the non-parametric Mann–Whitney U test employing the InsStat v3.0 software. Significance was accepted for *P* < 0.05.

**Table 2:** Primers used for quantification of *Trβ*, *Oc and Mgp* mRNA levels in *S. senegalensis*

Gene target	Primers	Sequence (5′-3′)	Base origin	Size (bp)
Sse UB	SseUB1	AGCTGGCCCAGAAATATAACTGCGACA	256	93
(AB291588.1)	SseUB2	ACTTCTTCTTGCGGCAGTTGACAGCAC	348	
SseTrβ	SseTrβF3	AAACAGAAGCGGAAGTTCCTGAGTGCAG	493	100
(GU946412)	SseTrβF4	CTTTGTTTCCTTCAGGTGTGTTTGCCATC	592	
Sse MGP	SseMGP1	GGTGCCCGGAGTCAGCCACA	88	113
(AY113679)	SseMGP2	AGCTGGTCTCTGGGGTCTCAGGTT	200	
Sse OC	SseOC1	GGTTCTCTGCTCCCTGGCCGTCCTCT	21	115
(AY823525)	SseOC2	GACGCCTGCTCCTGCTCCACAAACAA	135	

### In situ hibridization

To study the localization of *Trß*, *Oc and Mgp* gene expression patterns in different cell- types and tissues of the Senegalese sole, in situ hybridization was performed on histological sections of controls and malathion exposed fish. To synthesize correspondent probes, different PCR fragments around 93–135 bp belonging toTRß, OC and MGP cDNA, were amplified using specific primers (Table 2) and cloned with TOPO-TA Cloning kit (Invitrogen). Sense and anti-sense probes were synthesized using the DIG RNA labeling kit (SP_6_/T_7_) (Roche diagnostics, Indianapolis, USA) according to the manufacturer’s instructions. Specificity to these probes was confirmed by northern blot analysis. In situ hybridization protocols on histological sections from controls and malathion exposed Senegalese sole specimens were performed such as it is described by Ortiz-Delgado et al. [114]. Briefly, sections were rehydratedand permeabilised for 15 min in 10 μg/mL proteinase K in phosphatebuffered saline with Tween 20 (PBST: 137 mM NaCl, 2.7 mM KCl,1.8 mM KH_2_PO_4_, 10 mM Na_2_HPO_4_, 0.1% Tween-20) at 37 °C. After post-fixation in 4% paraformaldehyde-PBST for 30 min, sections were incubated for 2 × 15 min in PBST containing 0.1% active DEPC, and equilibrated for 15 min in PBST. The histological sections were then pre-hybridised for 2 h at 56 °C in the hybridization mix, and later, they were hybridised with the sense or antisense probes at 56 °C overnight in a humidified chamber. After incubation, the sections were washed three times in 2x SSC at 52 °C for 30 min, later they were washed twice at 52 °C with 1.4x SSC/0.6% CHAPS (3-[3-cholamidopropyl)-dimethylamino]1-propanesulfonate, and once with 1:1 PTW: maleic acid buffer (0.1 M maleic acid, 0.15 M NaCl, pH 7.5). Detection of the hybridized probe was carried out using alkaline phosphatase-coupled *anti*-digoxigenin antibody, and the hybridization signals were detected by NBT/BCIP systems according to the manufacturer’s instructions (Roche), adding 5 mM levamisole to neutralize endogenous alkaline phosphatase. The controls included hybridization with sense probes, RNase treatments before hybridization and absence of antisense RNA probe/anti-DIG antibody in the incubation medium.

### Statistical analysis

Comparison between groups were made by using one-way analysis of variance (ANOVA). Normality was checked using the Shapiro-Wilk’s test and the homogeneity of variances with the Levene’s test. Tukey’s post hoc test was used for identification of the statistically distinct groups. The computer program InStat v3.0. vas used to perform the analyses. Differences were considered statistically significant at *p* < 0.05. For statistical evaluation and graphical representations, control and solvent–carrier control were pooled, as they did not differ significantly with regard to the different recorded measurements.

The assessment for immunohistochemical results was performed independently by three observers, which analysed the same histological samples. From each sample, three histological slides of controls and of each experimental assay (1.56, 3.12 and 6.25 μg/L) were analysed from 4 dph onwards.

## Abbreviations

AA-AR: Alcian blue-alizarin red
AChE: Acetylcholinesterase.
DW: Dry weigh
ECDs: Endocrine disrupting chemicals
GCL: Ganglion cell layer
HPT: Hypothalamus-pituitary-thyroid axis
*I*_EM_: Index of eye migration
INL: Inner nuclear layer
IPL: Inner plexiform layer
LC_50_: Lethal concentration 50%
LOEC: Lowest observed effect concentration
MGP: Matrix-Gla-protein
NOEC: No observed effect concentration
OC: Osteocalcin
ONL: Outer nuclear layer
OP: Organophosphate pesticide
OPL: Outer plexiform layer
PCNA: Proliferating cell nuclear antigen
PL: Pigmentary layer
THs: Thyroid hormones
TL: Total length
TR: Thyroid receptor
